# Fat‐Containing Soft Tissue Lesions of the Limbs and Body Wall—A Pictorial Essay

**DOI:** 10.1111/1754-9485.70181

**Published:** 2026-09-07

**Authors:** Kai‐Zheong Lim, Cathryn Hui

**Affiliations:** ^1^ Monash Health Radiology Monash Health Clayton Victoria Australia; ^2^ School of Clinical Sciences Monash University Clayton Victoria Australia

**Keywords:** fat‐containing lesion, lipoma, lipomatous tumour, liposarcoma

## Abstract

Lipomatous tumours are frequently encountered on imaging. The spectrum of lipomatous tumours ranges from simple lipomas, which can be monitored clinically, to high‐grade liposarcomas, which require timely, multidisciplinary management. The presence of fat within lesions may also be observed in non‐lipomatous tumours, as well as in a variety of non‐neoplastic conditions. This pictorial review examines the key imaging characteristics of primary lipomatous tumours and other fat‐containing soft tissue lesions that can be found in the limbs and body wall.

## 1 Introduction

Fat‐containing soft tissue lesions are commonly seen in clinical practice and on imaging. The most frequently encountered fat‐containing lesions are lipomatous tumours, with lipomas representing almost 50% of all soft tissue tumours [1].

Fat content may also be seen in non‐lipomatous tumours and in a variety of non‐neoplastic disorders, due to fat entrapment or lipomatous differentiation. Fat may proliferate in synovium or leak from bone marrow when there is cortical breach.

In this pictorial review, we will examine the key imaging findings of these fat‐containing soft tissue lesions, with particular emphasis on MRI appearances (Table 1).

**TABLE 1:** An approach to fat‐containing soft tissue lesions of the limbs and body wall.

Lesion Characteristics	Differential Diagnosis
Simple‐appearing fatty lesions of the limbs and body wall – Isointense to subcutaneous fat – Few, thin, internal septations – < 10 cm in size	Lipoma ◦ Predominantly in superficial tissue of back, neck and extremities Hibernoma ◦ Predominantly in the thigh or shoulder ◦ Middle‐aged patients ◦ CT PET FDG avidity Neural lipomatosis ◦ Most commonly in median nerve ◦ Characteristic co‐axial cable appearance Lipoblastoma ◦ Rare paediatric tumour < 3 years old ◦ Rapidly enlarging ◦ PLAG1 mutation on cytogenetic testing
Large or complex‐appearing fatty lesions of the limbs and body wall Biopsy indications – Any fatty lesion > 10 cm – Atypical features ◦ Thick septations ◦ Nodular soft tissue foci ◦ Heterogeneous signal – Any recurrent lipoma after treatment	Lipoma ◦ As above ◦ Atypical features reported in 30% of lipomas due to necrosis/infarction ◦ No MDM2 amplification Hibernoma ◦ As above ◦ Immunohistochemical testing staining positive for S‐100 and negative for desmin Lipoblastoma ◦ As above Atypical lipomatous tumour/Well‐differentiated liposarcoma ◦ MDM2 amplification Liposarcoma ◦ Large and deep in location ◦ MDM2 amplification ◦ Marked T2 hyperintensity in myxoid subtype Massive localised lymphedema ◦ Middle‐aged, morbidly obese ◦ Large pedunculated soft tissue swelling of inner thigh ◦ Mixed signal including areas of fat, oedema and dystrophic calcifications Fat necrosis ◦ Variable imaging appearances ◦ May be associated with trauma ◦ Frequently occurs within lipomatous tumours
Predominant soft tissue lesions of the limbs and body wall with fatty elements	Liposarcoma ◦ As above ◦ May pose diagnostic challenges due to relative lack of adipose tissue Massive localised lymphedema ◦ As above Elasto fibroma dorsi ◦ > 40 years of age, female preponderance ◦ Infrascapular in location ◦ Bilateral in 50% of cases Tendon Xanthoma ◦ Associated with hypercholesterolemia ◦ Predominantly in Achilles tendon, hands and elbow ◦ Bilateral and symmetrical Vascular malformation ◦ High flow lesions: Fat may be interspersed between abnormal leash of vessels ◦ Low flow lesions: Fat seen between lobulations of a T2 hyperintense, enhancing lesion which may contain phleboliths Diffuse tenosynovial giant cell tumour ◦ T1 and T2 hypointense frond‐like projections due to hemosiderin deposits; T1 hyperintense signal fat foci occasionally seen
Fat within Fluid	Lipoma Arborescens ◦ Frond‐like lipomatous proliferations of synovium projecting into joint fluid ◦ Predominantly in knees; tendon sheaths and bursae may also be affected Intra‐articular fracture ◦ Lipohaemarthrosis due to escape of fat and blood from the marrow ◦ Three distinct layers: Fat, serum/synovial fluid and red blood cells Osteomyelitis ◦ Fat‐fluid levels may be seen adjacent to infected bone, indicating cortical breach ◦ May be intra‐ or extra‐articular Morel‐Lavallée lesions ◦ Shearing injury with disruption of the vascular and lymphatic channels, perforating the layers of soft tissue ◦ Collections of blood, lymph and fatty debris

## 2 Lipomatous (Adipocytic) Tumours

Lipomatous tumours are the most common type of soft tissue tumours of the extremities. The 2020 WHO Classification of Soft Tissue and Bone Tumours, 5th Edition, characterises lipomatous tumours according to the degree of lipomatous differentiation [2]. The benign category includes several types of lipomas. The intermediate category includes atypical lipomatous tumours and the malignant category encompasses various types of liposarcomas.

### 2.1 Benign Lipomatous Tumours

#### 2.1.1 Lipomas

Lipomas are benign tumours that contain mature adipose tissue [3]. Most lipomas are located within the superficial soft tissues of the back, neck, and extremities. On MRI, lipomas are usually well‐circumscribed masses, isointense to subcutaneous fat, with few, thin internal septations and minimal enhancement [3]. Intramuscular lipomas may interdigitate with skeletal muscle, which may be misinterpreted as thick septations (Figure 1). Nodular foci or septa thicker than 2 mm have been reported in 30% of lipomas. Lipomas may undergo necrosis or infarction, producing signal heterogeneity or mass‐like regions of soft tissue. These features overlap with those of liposarcomas [3, 4]. In such cases, biopsy is recommended. Immunohistochemical tests, in particular, fluorescent in situ hybridisation (FISH) for MDM2‐amplification, allow definitive diagnosis.

**FIGURE 1:**
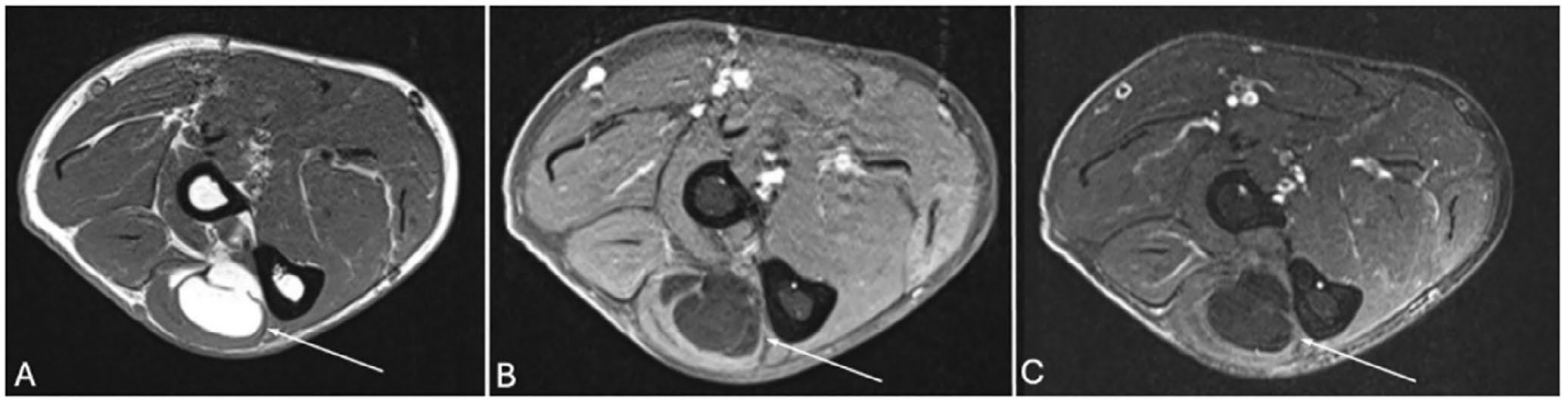
A 56‐year‐old man with a forearm intramuscular lipoma. Axial T1 (A), post‐contrast T1 fat‐saturated (B) and T2 fat‐saturated (C) MRI sequences demonstrate a well‐marginated T1 hyperintense lesion (arrow) within the extensor carpi ulnaris muscle interdigitating with muscle fibres, which resembles a septation.

#### 2.1.2 Hibernomas

Hibernomas are tumours of brown fat, termed due to the similarity to the fat in hibernating animals [5]. Hibernomas are usually found in the subcutaneous tissue of the thigh, shoulder, and neck regions [4]. On MRI, hibernomas are well‐defined fatty lesions. Internal T1‐ and T2‐hypointense septa and prominent vasculature are common. Characteristically on FDG‐CT PET, they demonstrate FDG avidity due to hypervascularity and intense glucose metabolism of their brown fat content [4, 5].

#### 2.1.3 Fibrolipomatous Hamartoma/Neural Lipomatosis

Fibrolipomatous hamartomas represent lipomatous proliferation within a peripheral nerve, with the median nerve most commonly affected [6]. On MRI, there is a ‘coaxial cable’ appearance, with serpiginous low T1‐signal intensity nerve fascicles surrounded by T1‐hyperintense fatty tissue, resulting from fat accumulation around nerve fascicles with separation of the individual nerve bundles (Figure 2).

**FIGURE 2:**
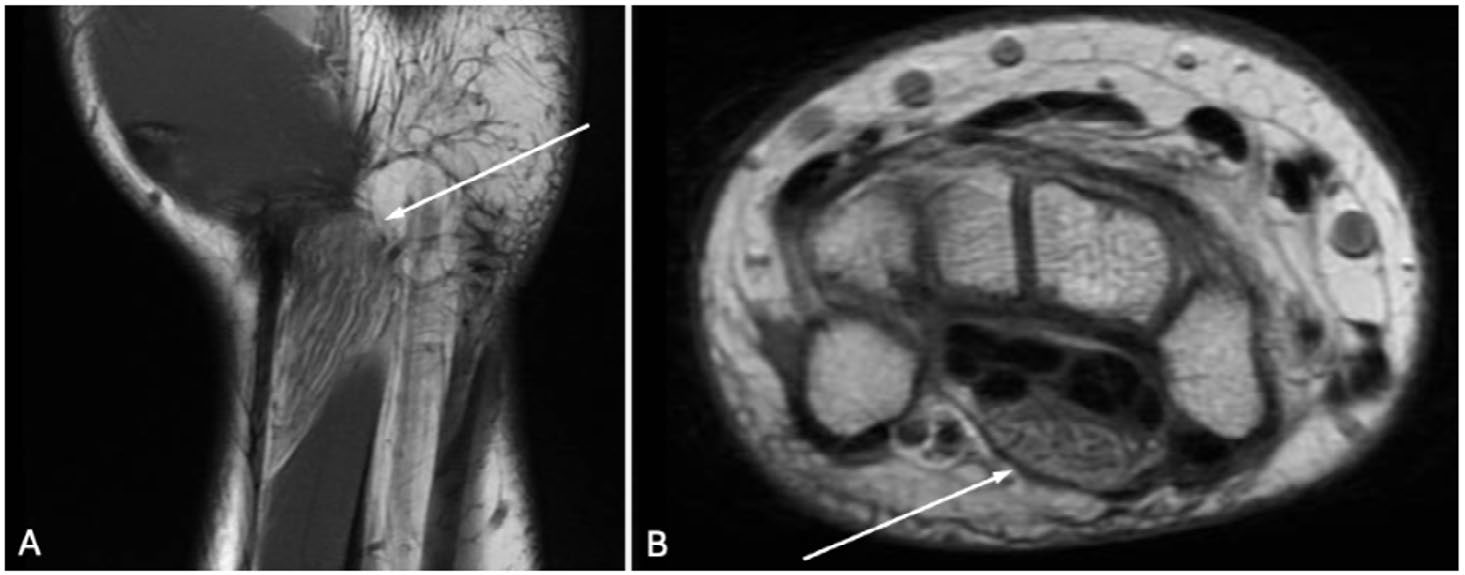
A 16‐year‐old female with lipomatosis of the median nerve. Coronal T1 (A) and axial proton‐density (B) MRI images of the left wrist demonstrate the pathognomonic ‘co‐axial cable’ appearance with T1 hyperintense fat surrounding and separating the nerve bundles in an enlarged median nerve.

#### 2.1.4 Spindle Cell Lipoma and Pleomorphic Lipomas

Spindle cell lipomas typically present as well‐defined superficial complex fatty masses involving the shoulder, posterior neck, and upper back [7, 8]. Pleomorphic lipomas represent a further lipoma variant. The imaging appearances can be variable due to differing amounts of lipomatous and non‐lipomatous components [9].

#### 2.1.5 Lipoblastomas

Lipoblastomas are rare childhood soft‐tissue tumours, predominantly arising in extremities as smooth, rapidly enlarging masses [10]. Their signal characteristics may be variable depending on the proportions of fat and myxoid stroma.

### 2.2 Intermediate Lipomatous Tumours

#### 2.2.1 Atypical Lipomatous Tumours (ATL) and Well Differentiated Liposarcoma (WDL)

ALTs and WDLs share similar histological and molecular findings. The term atypical lipomatous tumour is reserved for locally aggressive tumours at sites in which complete surgical resection is possible, such as those in the soft tissues of the limbs and in superficial locations [3, 4]. Well‐differentiated liposarcomas refer to deep lesions, such as in the retroperitoneum and mediastinum [11]. On imaging, ALT/WDLs are suspected if there are thick septa > 2 mm and soft tissue nodules (Figure 3) [12, 13]. The imaging features overlap with complicated lipomas. Molecular analysis with FISH demonstrating MDM2‐amplification confirms a diagnosis of ALT/WDL.

**FIGURE 3:**
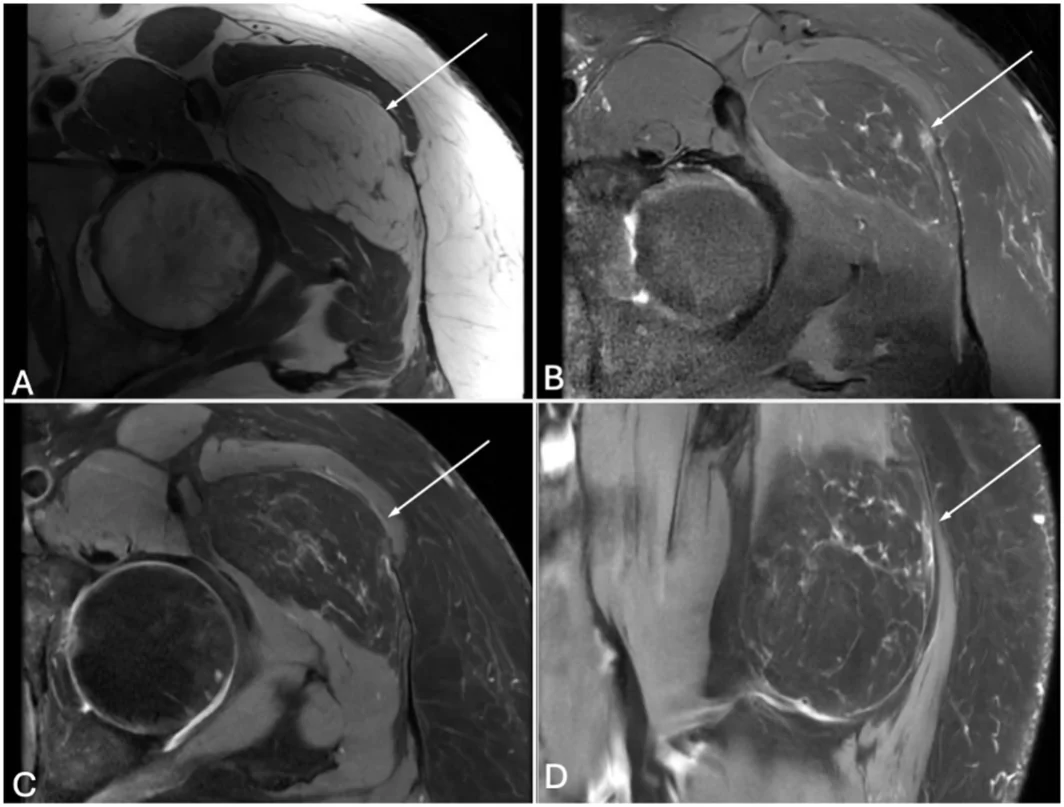
A 48‐year‐old male with atypical lipomatous tumour anterior to the left hip confirmed on FISH testing with MDM2 amplification. Axial T1 (A), T2 fat‐saturated (B) MRI sequences show a fatty mass deep to the tensor fascia‐lata with thick internal septations and small areas of mildly T2 hyperintense soft tissue, associated with enhancement, illustrated on axial (C) and coronal (D) post‐contrast T1 fat‐saturated sequences.

### 2.3 Malignant Lipomatous Tumours

#### 2.3.1 De‐Differentiated Liposarcomas

Approximately 10% of well‐differentiated liposarcomas progress to de‐differentiated liposarcomas (DDLs), which have the potential for metastases [3]. DDLs contain non‐fatty areas (Figure 4). They are frequently large and deep in location. These lesions demonstrate MDM2 amplification on FISH. MDM2 amplification FISH testing is recommended for lipomatous tumours greater than 10 cm without atypia, for any sized lesion with borderline atypia and for recurrence of treated lipomas [3].

**FIGURE 4:**
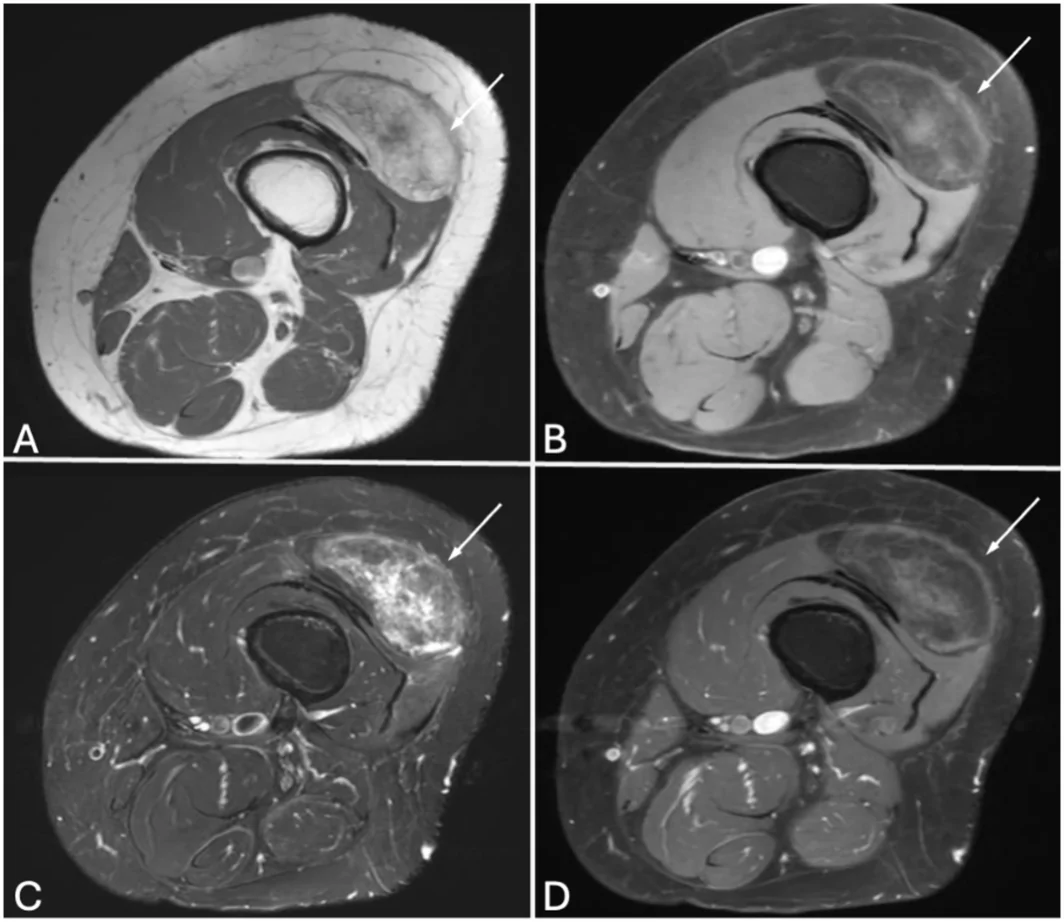
A 60‐year‐old female with dedifferentiated liposarcoma of the thigh. Axial T1 (A), T1 fat‐saturated (B), T2 fat‐saturated (C), post‐contrast T1 fat‐saturated (D) MRI images showing a large heterogeneous fatty mass in the rectus femoris and vastus lateralis muscles with thickened septations and poorly defined nodular areas of soft tissue signal.

#### 2.3.2 Myxoid Liposarcoma

Liposarcomas of myxoid subtype contain gelatinous myxoid tissue [14]. On MRI, these lesions show marked T2 hyperintensity due to the myxoid component, with T1‐hyperintense linear or nodular areas of lesional fat [4]. The lipomatous content, however, may be minimal and not readily identifiable on imaging (Figure 5) [3].

**FIGURE 5:**
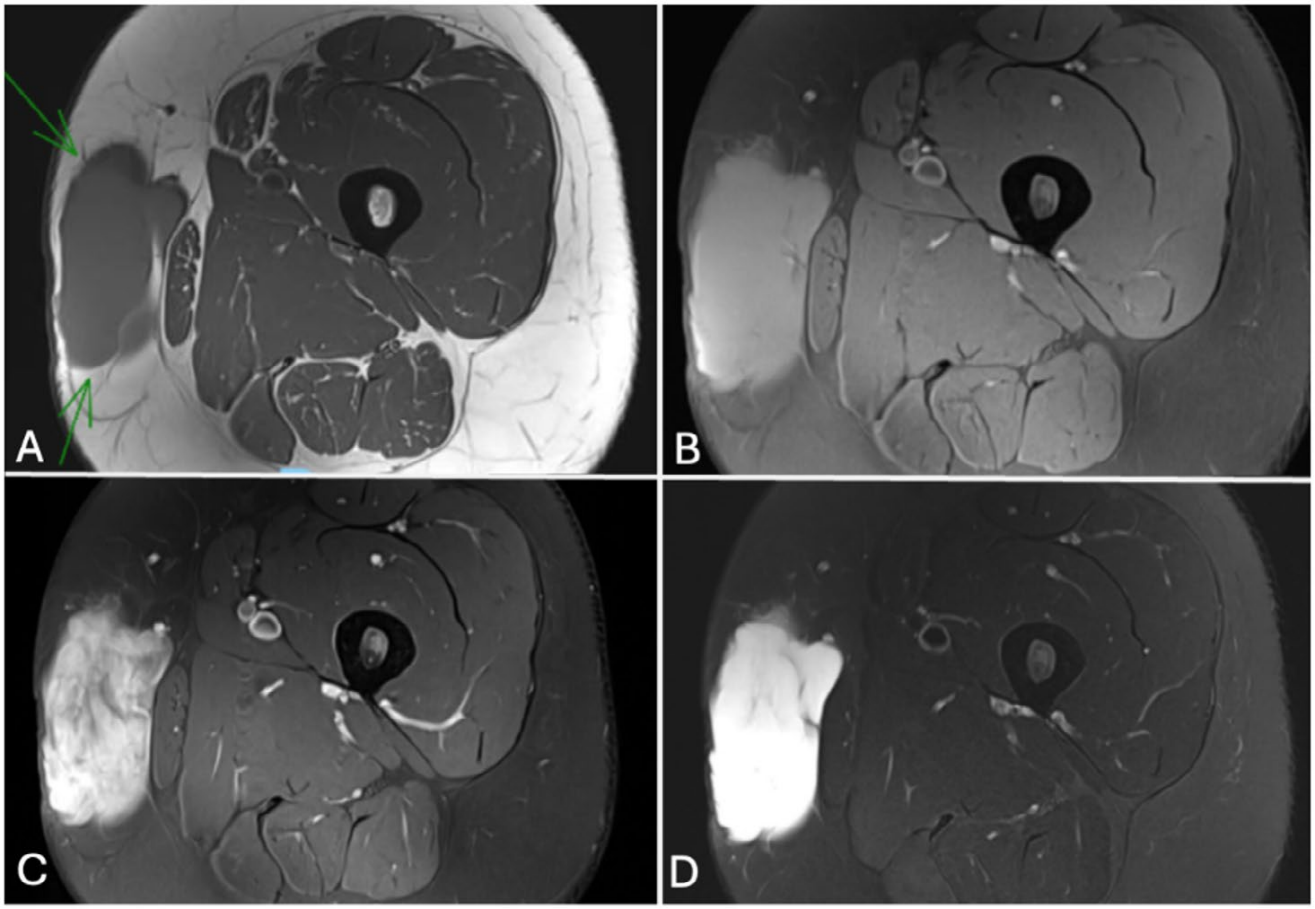
A 24‐year‐old female with myxoid liposarcoma of the thigh. Axial T1 (A), T1 fat‐saturated (B), T2 fat‐saturated (C), post‐contrast T1 fat‐saturated (D) MRI images demonstrate a lobulated mass in the subcutaneous fat with heterogeneous T2 hyperintensity reflecting myxoid matrix. Fat signal may be difficult to detect in these lesions when fat content is scant.

### 2.4 Non‐Lipomatous Tumours Containing Fat

#### 2.4.1 Elastofibroma Dorsi

Elastofibroma dorsi is a benign soft tissue fibroblastic tumour that classically occurs in the infrascapular region. These lesions occur in patients after the 4th decade of life with a predilection for females [15]. On MRI, the soft tissue component of the lesions is isointense to skeletal muscle with T1‐hyperintense internal fat striations, although there may be minimal fat content (Figure 6).

**FIGURE 6:**
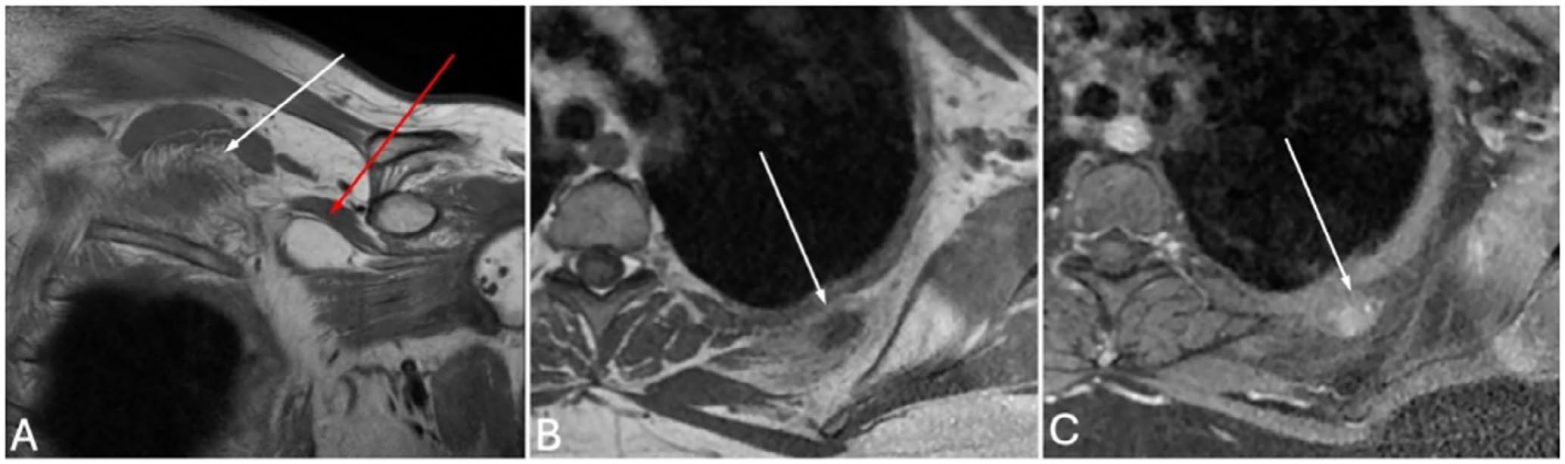
A 48‐year‐old male with an elastofibroma dorsi and adjacent lipoma. Coronal T1 (A), axial T1‐fat‐saturated (B), post‐contrast T1‐weighted fat‐saturated (C) MRI images demonstrate an elastofibroma dorsi with predominant signal similar to skeletal muscle. There are striated internal areas of fat signal and a mild degree of heterogeneous enhancement (white arrow). Incidental subscapularis intramuscular lipoma is also noted (red arrow).

#### 2.4.2 Tendon Xanthomas

In familial hypercholesterolaemia, there is a genetic predisposition for increased deposition of plasma low‐density lipoprotein cholesterol [16]. Intratendinous cholesterol deposits lead to tendon xanthomas, particularly occurring in the Achilles tendon, hands and elbow. On MRI, xanthomas demonstrate intermediate‐T1 and T2 signal with internal streaks of fat signal, which result in a striated appearance along the plane of the tendon or a speckled appearance in cross‐section (Figure 7).

**FIGURE 7:**
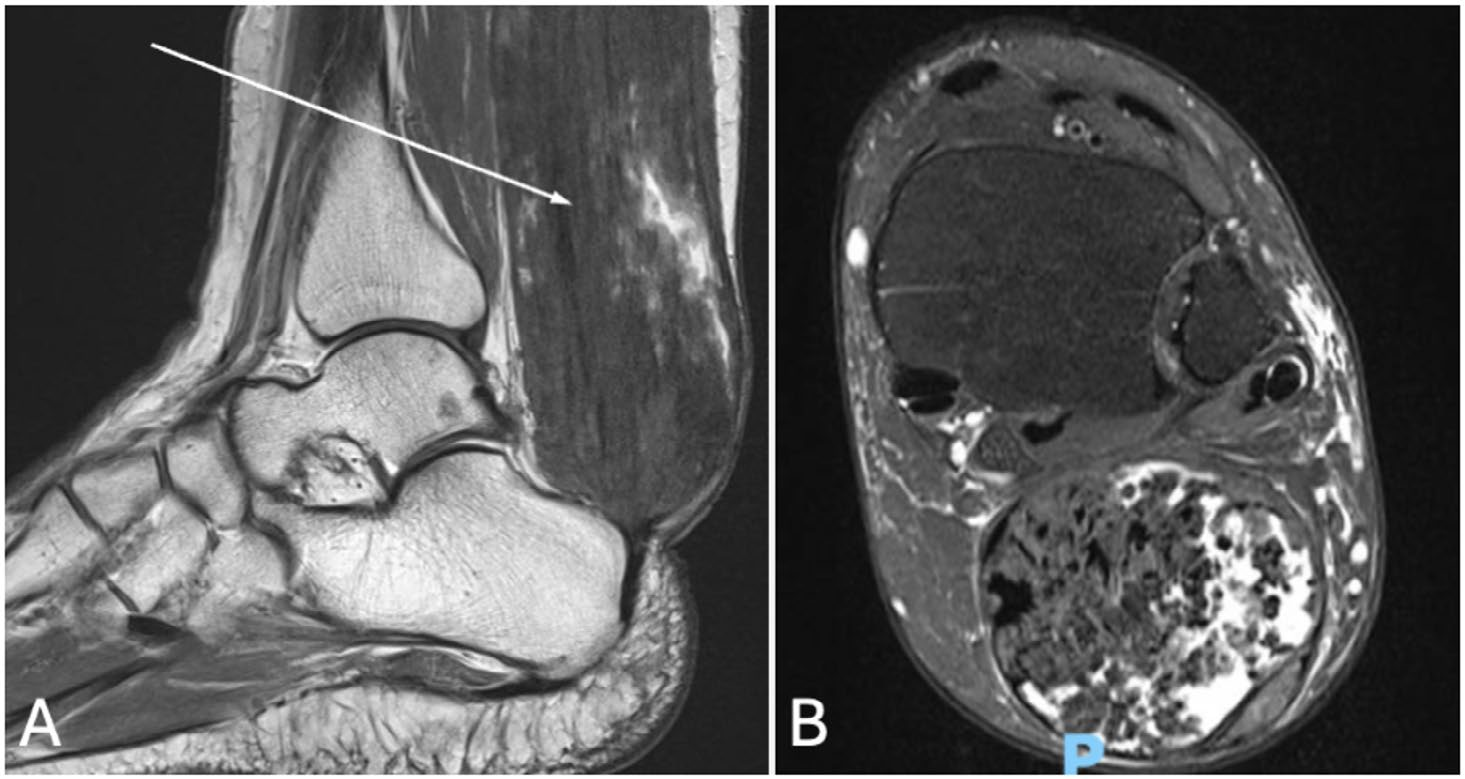
A 55‐year‐old female with an Achilles tendon xanthoma. Sagittal proton‐density (A) and axial T2 fat‐saturated (B) MRI sequences demonstrate marked enlargement of the Achilles tendon with striated (A) and speckled (B) appearances of fat within the xanthoma (arrow).

#### 2.4.3 Diffuse Tenosynovial Giant Cell Tumours

Diffuse tenosynovial giant cell tumours (DTGCT) are characterised by villous proliferation of the synovium. Variable amounts of fibrovascular elements and hemosiderin are present; the latter results in T2‐hypointensity of the frond‐like projections. (Figure 8). In addition, fat may also be observed within the synovial proliferation [17].

**FIGURE 8:**
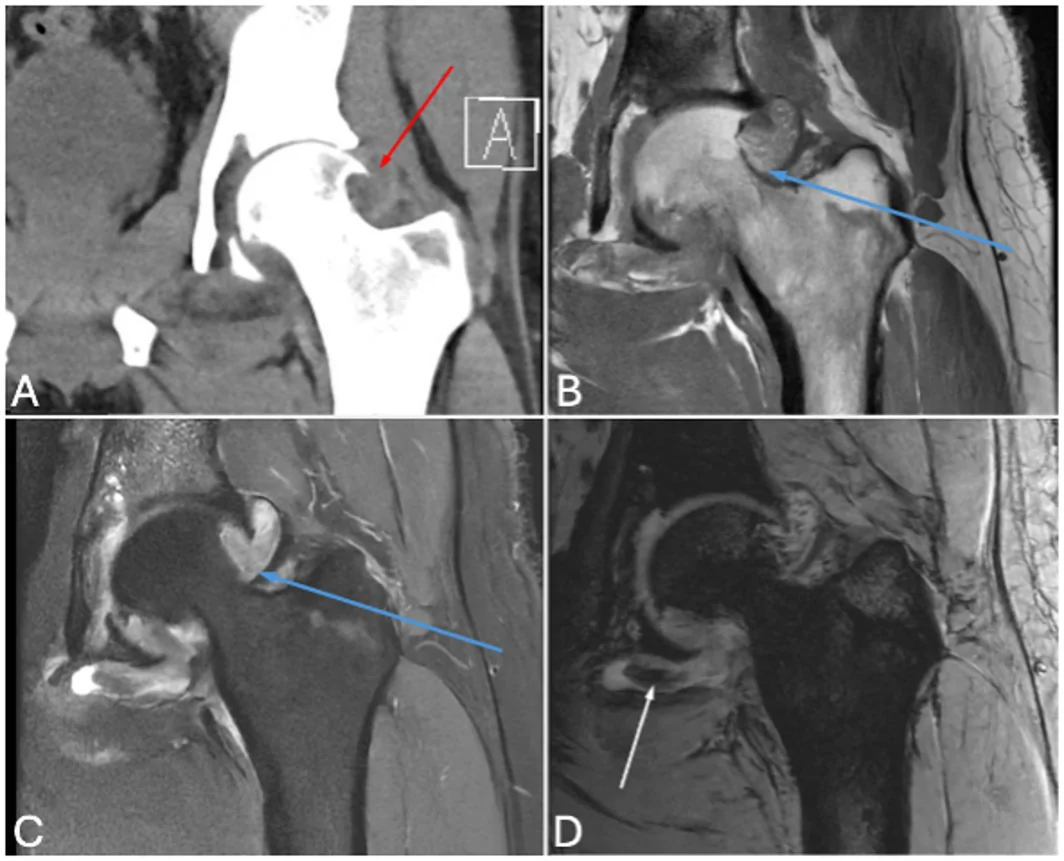
A 31‐year‐old male with tenosynovial giant cell tumour of the hip containing prominent fat. Coronal CT image (A) demonstrates areas of fatty attenuation within the joint and well‐defined erosion at the lateral femoral head–neck junction (red arrow). Coronal T1 (B), T2 fat‐saturated (C) and gradient‐echo (D) MRI sequences demonstrate fat signal in the synovium (blue arrow). Susceptibility on the gradient‐echo sequences reflects hemosiderin deposition (white arrow).

#### 2.4.4 Rare Fat‐Containing Non‐Lipomatous Tumours

Rare soft tissue lesions that may contain fat include benign lipoblastic neural tumours, lipofibromatosis, and phosphaturic mesenchymal tumours (PMT). PMTs are benign tumours associated with reduced phosphate resorption [18, 19], resulting in oncogenic osteomalacia.

### 2.5 Non‐Neoplastic Fat Containing Lesions

#### 2.5.1 Vascular Malformations

Vascular malformations may contain fat between and around abnormal vessels, which can mimic a fat‐containing vascular mass. In the diffuse subtype of venous vascular malformation, fat hypertrophy may be associated with fatty replacement of muscle and muscle atrophy (Figure 9). Fibro‐adipose vascular anomaly (FAVA) is a rare vascular malformation surrounded by fibroadipose tissue within skeletal muscle [20].

**FIGURE 9:**
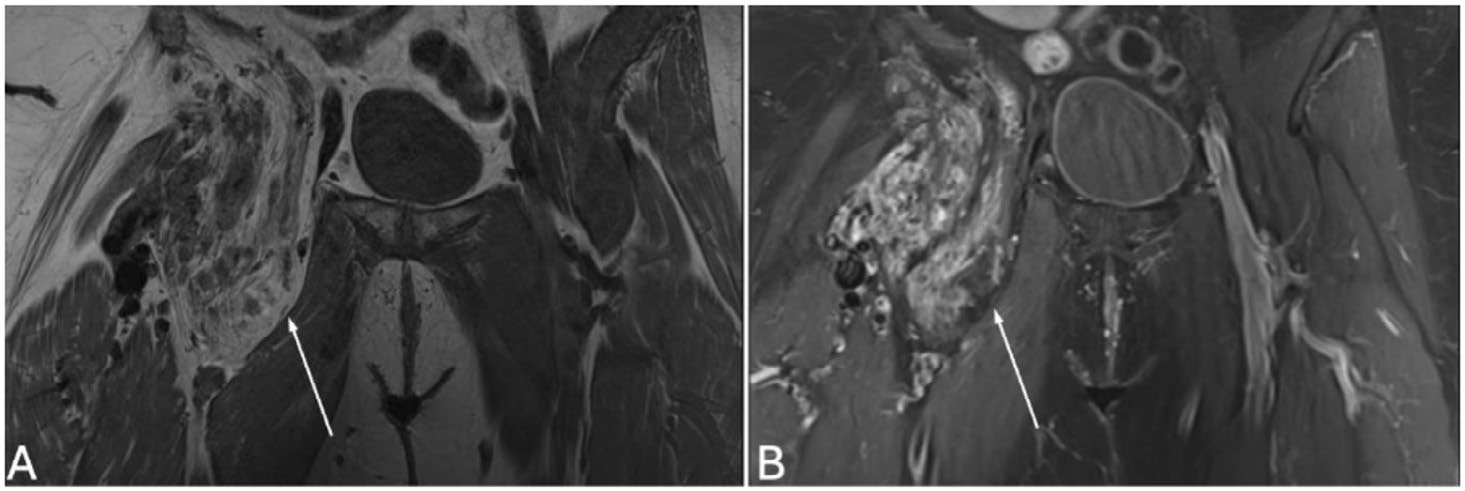
A 34‐year‐old female with a right inguinal high‐flow arteriovenous malformation containing prominent internal fat signal. Coronal T1 (A) and post‐contrast T1 fat‐saturated (B) MRI sequences demonstrate serpiginous areas of enhancement of the vessels with interspersed fat signal (arrow) and fatty replacement of adjacent musculature.

#### 2.5.2 Massive Localised Lymphedema

Massive localised lymphedema occurs in middle‐aged, morbidly obese adults and presents as a tumefactive pseudo‐lesion. It is characterised histologically by fibrosis, dilated lymphatics, and fat necrosis [21]. The aetiology is thought to be related to impaired lymphatic flow. The inner thigh is the most common site affected. On imaging, massive localised lymphedema manifests as a large pedunculated mass‐like soft‐tissue swelling exhibiting intermediate‐T1 signal with multiple septations, as well as areas of oedema and T1 hyperintense fat (Figure 10).

**FIGURE 10:**
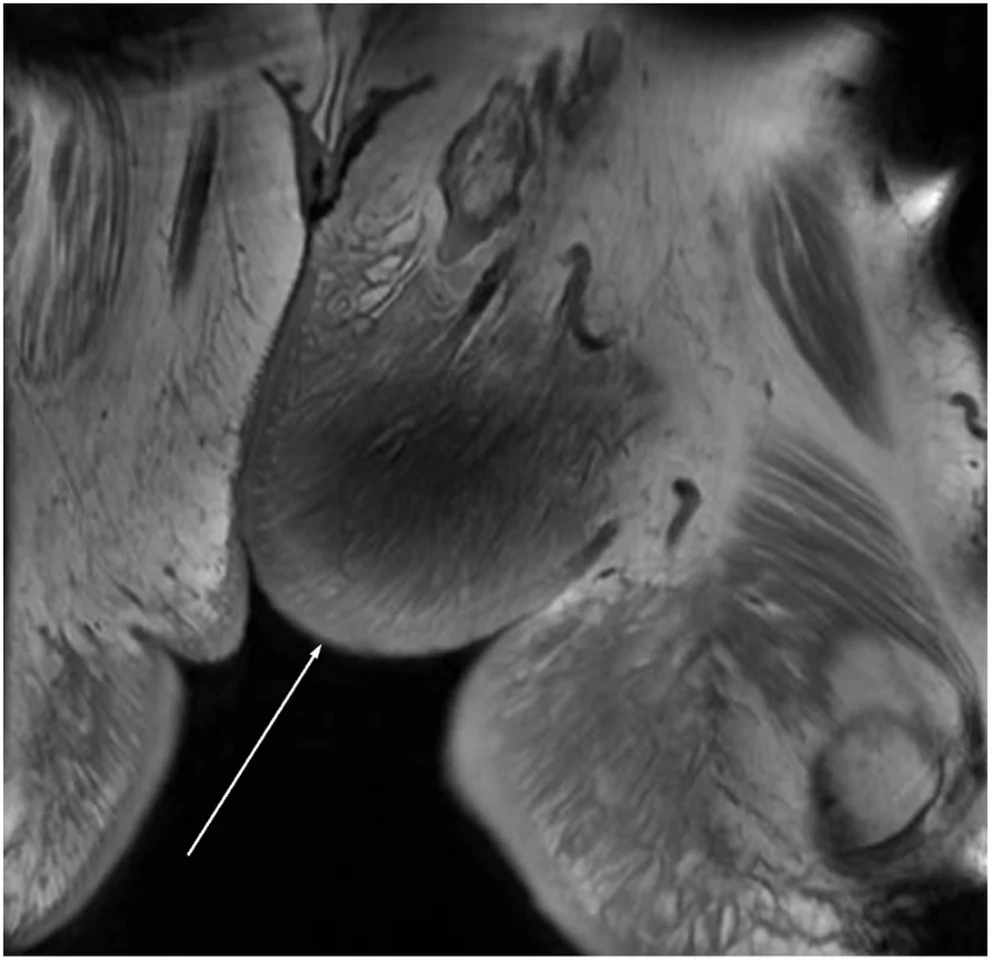
A 52‐year‐old morbidly obese male with massive localised lymphedema in the proximal medial thigh. Coronal T1‐weighted MRI sequence demonstrates streaky areas of intermediate and high T1 signal within a pedunculated fatty pseudo‐mass (arrow).

#### 2.5.3 Lipoma Arborescens

Lipoma arborescens is attributed to replacement of subsynovial tissue by mature fat cells. The suprapatellar pouch of the knee is the most commonly involved joint [22]. The primary form is idiopathic, and the secondary form occurs as a reaction to chronic inflammation. On MRI, the characteristic findings include frond‐like fatty signal arising from the synovium, usually associated with a joint effusion (Figure 11).

**FIGURE 11:**
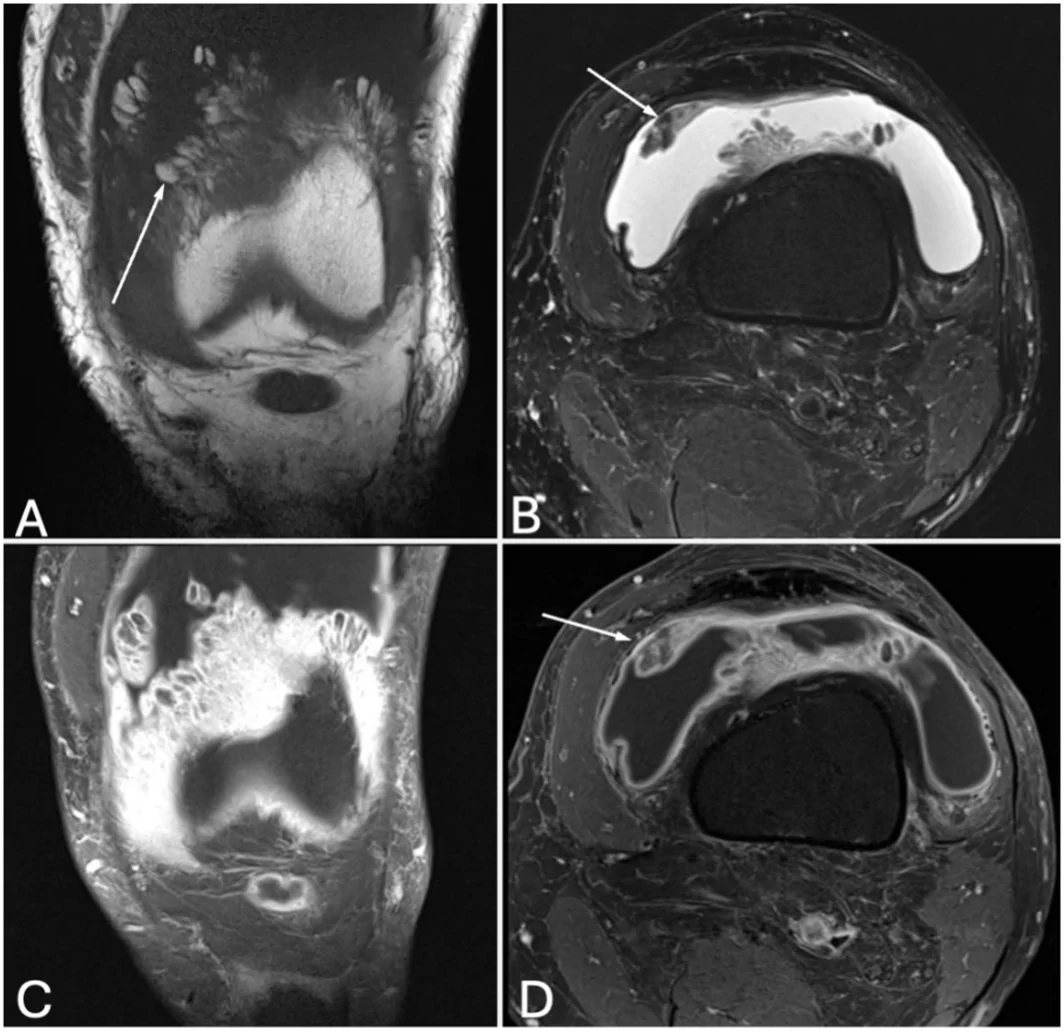
A 69‐year‐old male with lipoma arborescens of the knee. Coronal T1 (A), axial T2 fat‐saturated (B), coronal (C) and axial (D) post‐contrast T1 fat‐saturated MRI sequences demonstrate frond‐like areas (arrow) of fat signal along the synovial lining of the suprapatellar recess associated with synovial enhancement and a moderate joint effusion.

#### 2.5.4 Lipohemarthrosis

Layering of fluid and fat in a joint most frequently results from an intra‐articular fracture. On MRI, three layers may be visible: A superficial layer of fat, a middle layer comprised of serum/synovial fluid, and a deep layer formed by red blood cells. Within the first two hours after a fracture, intramedullary fat leaks into the joint cavity. Fat globules form, producing an undulating fat‐fluid interface before evolving into the typical straight fat‐fluid level [23]. Fat‐fluid levels may also be seen in the setting of osteomyelitis, indicating cortical breach [24].

#### 2.5.5 Morel‐Lavallée Lesions

Morel‐Lavallée lesions are closed traumatic soft‐tissue degloving injuries, characterised by separation of the hypodermis layer from the underlying fascia due to a shearing force. Vascular and lymphatic channels are disrupted, resulting in collections of blood, lymph and fatty debris. On MRI, well‐defined collections may be encapsulated and extend along the plane of the fascia. Fluid–fluid levels, septations and fatty foci may be seen. (Figure 12).

**FIGURE 12:**
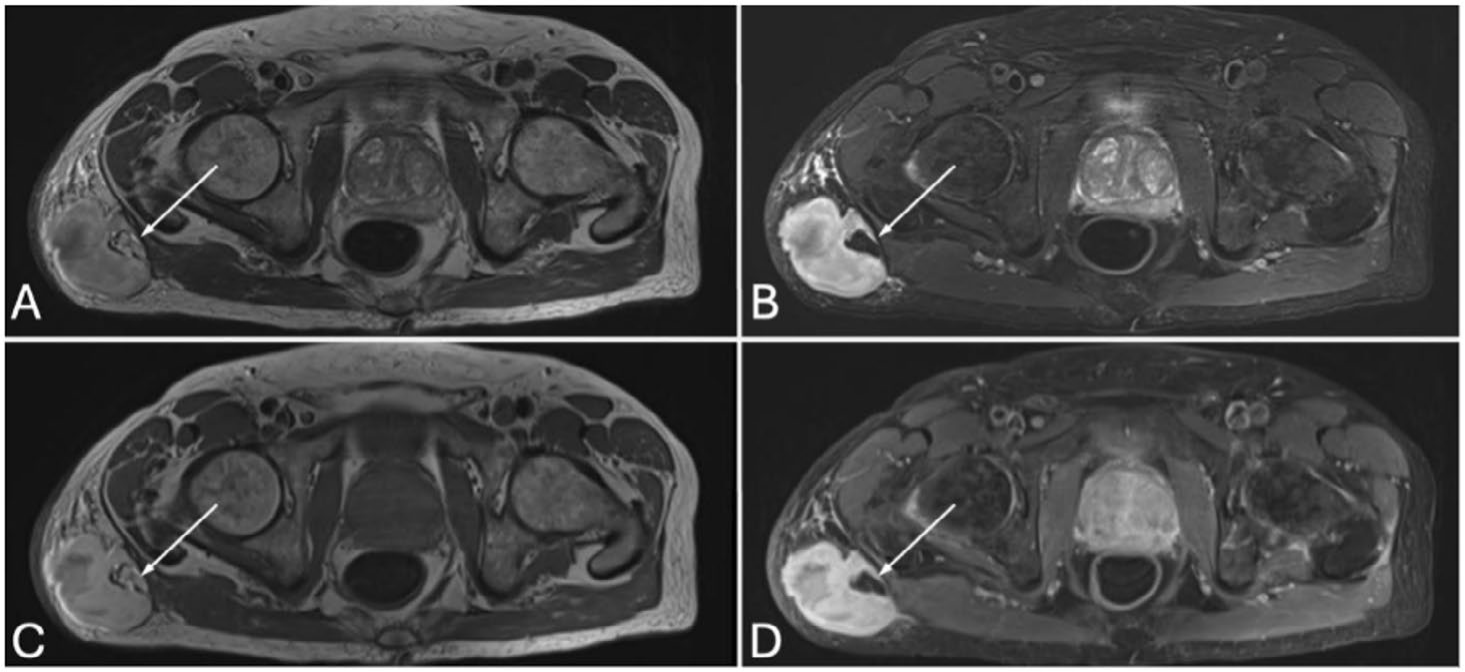
A 78‐year‐old man with traumatic Morel‐Lavallée lesion overlying the right lateral hip. T2 axial (A), T2 fat‐saturated (B), T1 axial (C) and post‐contrast T1 fat‐saturated (D) MRI images demonstrate a non‐enhancing T1‐ and T2‐hyperintense collection in the subcutaneous fat, along the fascia overlying the greater trochanter, containing a globule of fat (arrow).

#### 2.5.6 Fat Necrosis

Fat necrosis most often presents as a soft‐tissue mass over bony prominences [26]. There may be a history of local trauma. Imaging appearances can be variable; however, fat necrosis is most commonly described as a globular lesion with T1 hyperintense fat signal, an irregular rim of T1 hypointensity with variable enhancement. Fat necrosis may occur within lipomas, which may resemble features of liposarcoma [12, 25].

## 3 Conclusion

Familiarity with the imaging appearance of the spectrum of fat‐containing lesions in soft tissues will assist in diagnosis and may prevent unnecessary intervention. In cases of uncertainty, a multidisciplinary approach, including biopsy and molecular testing, assists in diagnosis and planning treatment.

## Author Contributions

**Kai‐Zheong Lim:** writing – review and editing, writing – original draft. **Cathryn Hui:** conceptualization, writing – review and editing, supervision, writing – original draft.

## Funding

The authors have nothing to report.

## Ethics Statement

The authors have nothing to report.

## Conflicts of Interest

The authors declare no conflicts of interest.

## Data Availability

Data sharing not applicable to this article as no datasets were generated or analysed during the current study.
